# Case report: Nutritionally supported perioperative chemo-immunotherapy for advanced gastric cancer with incomplete pyloric obstruction

**DOI:** 10.3389/fonc.2024.1383076

**Published:** 2024-04-23

**Authors:** Mi Jian, Zhensong Yang, Xue Hu, Xixun Wang, Zhenbin Zhang, Yifei Zhang, Xinna Song

**Affiliations:** ^1^ Department of Gastrointestinal Surgery, Yantai Yuhuangding Hospital Affiliated to Medical College of Qingdao University, Yantai, Shandong, China; ^2^ Department of Nutrition Department, Yantai Yuhuangding Hospital Affiliated to Medical College of Qingdao University, Yantai, Shandong, China

**Keywords:** nutritional intervention, gastric cancer, pyloric obstruction, immunotherapy, perioperative treatment

## Abstract

This case describes the benefits of perioperative chemo-immunotherapy for advanced gastric cancer and incomplete pyloric obstruction, supplemented with nutritional support. Early parenteral nutrition to stabilize nutritional status and mitigate nutrition impact symptoms, and in addition, throughout the chemo-immunotherapy perioperative period also maintained oral nutrition support and a tailored dietary plan. Above nutritional support maintained the patient’s physical condition during immunotherapy. Eventually, this combination therapy plan leads to a partial response. On the other hand, a combination of therapies that focus more on immune checkpoint inhibitor may be able to mitigate the side effects of chemotherapy. Such findings may yield novel prospects for patients with advanced gastric cancer and incomplete pyloric obstruction, enabling them to achieve better outcomes.

## 1 Introduction

Gastric cancer (GC) ranks among the top five malignant tumors worldwide in terms of incidence and mortality, with most patients were diagnosed with advanced cancer (1). In patients with advanced gastric cancer, obstruction is a common accompanying symptom, and surgery remains a critical treatment for patients with obstruction. However, high recurrence rate and elevated perioperative complication risk were observed in patients of advanced gastric cancer with obstruction accepting radical operation sequential adjuvant therapy due to poorly controlled tumor burden and inferior physical condition (2, 3). For such patients, perioperative therapy may significantly extend the survival of patients with gastric cancer, but nutrient intake disorders often limit the use of perioperative therapy. Therefore, nutritional therapy is necessary for some patients receiving perioperative therapy.

Over the past decade, immune checkpoint inhibitors (ICIs) have emerged as a promising alternative to conventional systemic chemotherapy for selective advanced GC cohorts (4). Various ICIs have demonstrated considerable advantages for individuals with GC, approved by FDA bureau (4, 5). In addition, the overlooked nutritional status is critical to the success of immune-based combination therapy (6). In our case, both tumor progression and the toxic side effects of chemotherapy deteriorate nutrient intake, while immunotherapy offers the possibility of reducing this deterioration. Meanwhile, the addition of early nutritional intervention contributes to the success of perioperative chemo-immunotherapy.

In this case, a patient with advanced GC accompanying incomplete pyloric obstruction received short-term parenteral nutrition and oral nutrition support and a tailored dietary plan throughout the perioperative chemo-immunotherapy. The patient achieved a partial response (PR) and underwent radical surgery. This case suggests the potential of combination of perioperative chemo-immunotherapy and nutritional intervention for patients with advanced GC and incomplete pyloric obstruction.

## 2 Case

**Figure 1:**
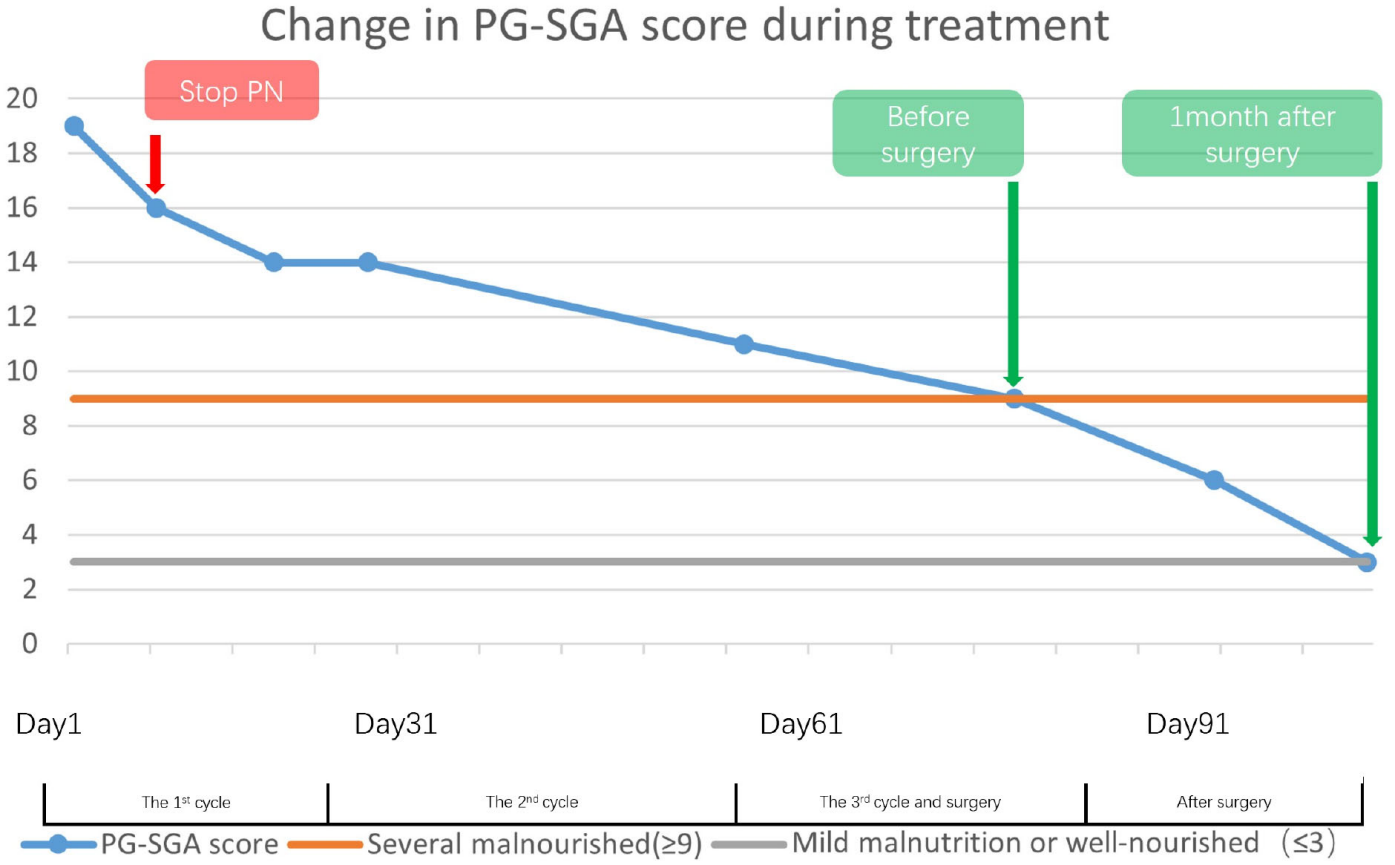
This illustrates the trajectory of patients’ PG-SGA scores from the first cycle of the treatment through to one-month post-surgery, indicating a progressive shift in nutritional status from severe to mild malnutrition. Despite the patient being severely malnourished before surgery, the marked reduction in scores throughout the course of treatment lends credence to the efficacy of the nutrition therapy.

A 62-year-old female patient was diagnosed with gastric adenocarcinoma at a local hospital and came to our hospital for better treatment outcomes. The patient experienced recurrent abdominal pain and vomiting over the past month, which progressively worsened over time. She did not have any other disease or a family history of cancer. Physical examination revealed tenderness in the upper abdomen but no palpable abdomen mass was detected. Upon admission, serum tumor marker test revealed a significant increase in alpha-fetoprotein (AFP) levels, carcinoembryonic antigen (CEA), carbohydrate antigen 125 (CA-125). PG-SGA score can provide a comprehensive assessment of her physical, anthropometric and functional condition, recent oral intake capacity and the subjective recall of nutrition impact symptoms which is assessed by our dieticians (7, 8). The nutritional assessment results indicated that she suffered from severe malnutrition (**Figure 1**).

### 2.1 The first cycle of perioperative treatment

**Figure 2:**
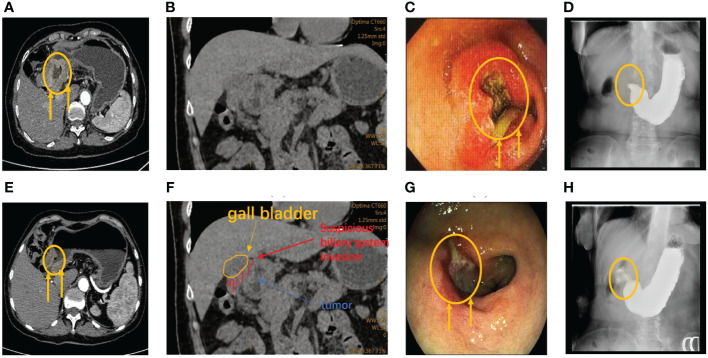
**(A)** CT reveals abnormal thickening of the antrum wall and incomplete obstruction prior to therapy. **(E)** Following three treatment cycles, CT scans showed significant improvement with disease regression, tumor retraction, and alleviated obstruction. **(B, F)** CT examination after admission showed suspicious biliary invasion. **(C)** Before therapy, gastroscopy revealed nearly complete stenosis of the pyloric ring cavity, accompanied by obstructive and edematous surrounding tissues. **(G)** Preoperative gastroscopy revealed obvious tumor retraction, along with significantly reduced obstruction and tissue edema compared to pretherapy gastroscopy. **(D, H)** Barium meal shows incomplete pyloric obstruction before the second cycle of treatment.

During the first multidisciplinary team (MDT) discussion, we diagnosed the patient had advanced gastric cancer with incomplete pyloric obstruction based on the imaging, endoscopic, and pathological evidence, and determined a clinical stage of cT4bN2M0 (**Figure 2**). At the beginning of our communication with this patient and her family, they chose surgery first. But, after we analyzed the current state of the disease and communicated with them about the risk of duodenal stump fistula (DSF) and high tumor mutation burden (TMB), they finally agreed to receive perioperative chemoimmunotherapy every 3 weeks first, consisting of intravenous oxaliplatin 200mg on day 1, oral tegafur–gimeracil–oteracil potassium (S-1) 60mg twice daily on days 1–14, oral Apatinib Mesylate Tablets 250mg on days 1-21 and intravenous Camrelizumab 200mg on day1 first.

**Figure 3:**
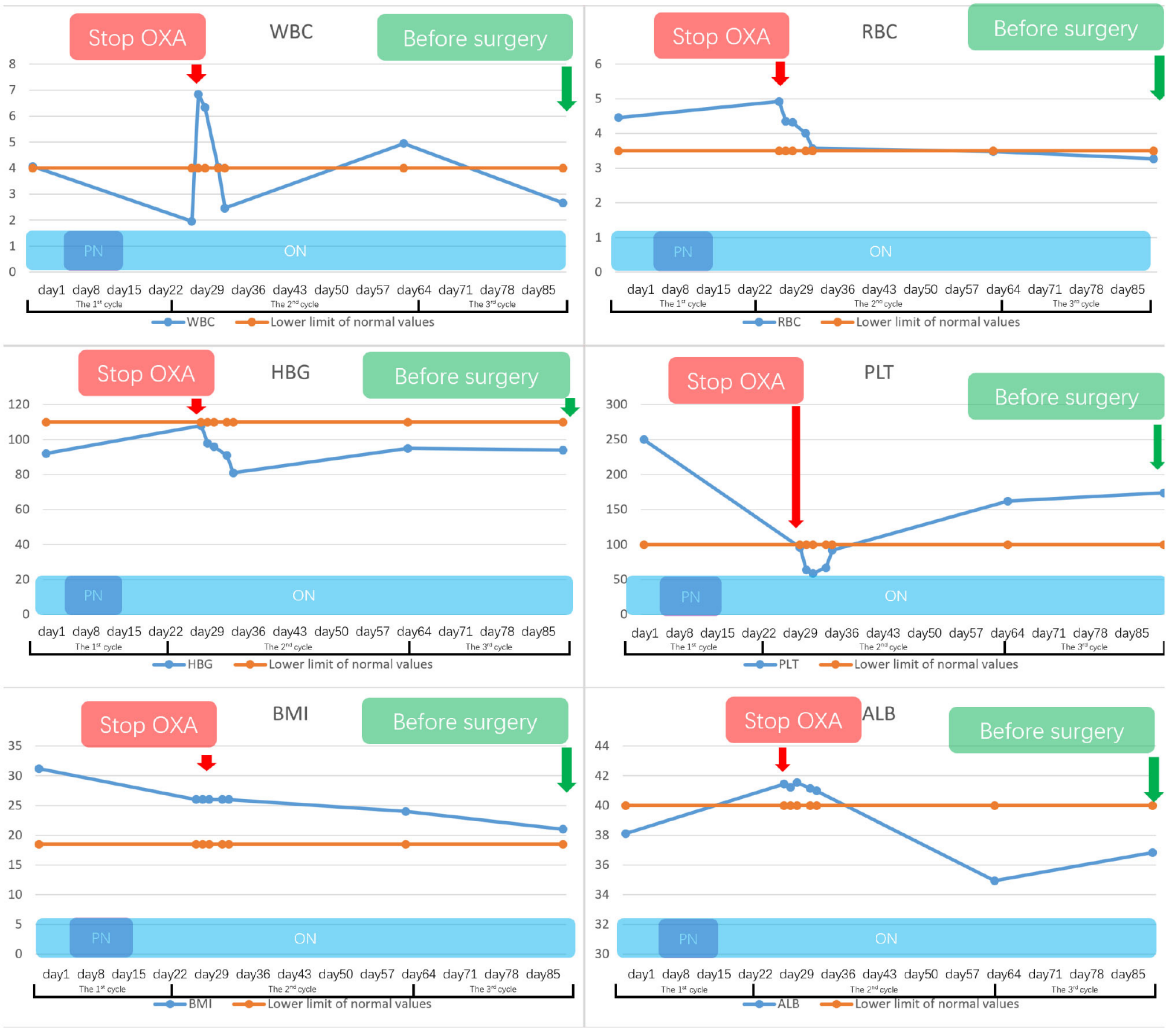
This study presents the variations in the patient’s nutritional examination indicators from admission to the preoperative period. With oral nutritional support, supplemented by parenteral nutrition and a tailored dietary plan, this patient’s nutritional indicators were generally maintained within the normal range. However, there was a notable occurrence of severe bone marrow suppression following the initial cycle of Oxaliplatin treatment, which subsided after discontinuing the medication. Notably, the patient did not exhibit any evident surgical contraindications based on the preoperative indicators.

Initially, the patient suffered significant side effects, including nausea and vomiting. Given the nutrition intake disorder stemming from incomplete obstruction and post-chemotherapy side effects of this patient, we adopted a combination regimen including parenteral nutrition (PN), oral nutrition support and a tailored dietary plan. Additionally, we continued to monitor various indicators of nutrition status, such as BMI, total protein, albumin, red blood cell, CRP levels, and hemoglobin (**Figure 3**).

### 2.2 The second cycle of perioperative chemo-immunotherapy

Before the second cycle of treatment, the imaging showed that the patient still suffered the incomplete pyloric obstruction (**Figure 2C, F**). And the blood tests showed a significant drop in her platelets and white blood cells (**Figure 3**). This patient and her family are concerned about whether subsequent treatment can be carried out and whether early surgery will have better results.

For the gastrointestinal adverse reactions and myelosuppression in the first cycle, our MDT team initially excluded the possibility of tumor progression according to the Barium meal results and attributed these side effects to the intravenous of oxaliplatin (**Figures 2**, **3**). Meanwhile considering the non-regressive state of the disease, we communicated with the patient and her family about the necessary of subsequent combination therapy for diminishing the TMB and the risk of DSF. Finally, we stop the use of oxaliplatin to alleviate adverse effects and gave the patient platelets transfusion and white blood cells raising drugs to treat the abnormalities of hemogram.

On the other hand, hemoglobin and albumin were significantly increased, and the PG-SGA scores decreased, indicating that nutritional therapy was effective (**Figures 1**, **3**). Given that the PG-SGA score is a comprehensive nutritional score that combines the patient´s physical, anthropometric and functional condition, recent oral intake capacity and the subjective recall of nutrition impact symptoms and is evaluated by professional physicians, we judge that the patient’s nutritional and physical condition was better than before treatment (7, 8). Subsequently, we discontinued PN and maintained the oral nutrition support and a tailored dietary plan.

All treatment options are used in consultation with the patient and her family.

### 2.3 The third cycle of perioperative chemo-immunotherapy

Before the third cycle of treatment, the patient could start eat semi-liquid diet which indicated the sign of tumor regression. Taking into account the improvement of the patient’s dietary status, we further reduced the proportion of oral nutrition support and instead increased the proportion of the tailored dietary plan. Moreover, the recovery from myelosuppression confirmed the correctness of stopping oxaliplatin (**Figure 3**). The patient then completed the third cycle of perioperative chemo-immunotherapy with an increasing food intake.

### 2.4 Perioperative management

After three cycles of treatment, the disease was reevaluated in the second MDT discussion. Imaging and endoscopic examinations showed that the tumor had shrunk significantly and the obstruction had been relieved (**Figure 2D, E**). The patient achieved PR during the treatment. Meanwhile, considering that the physical condition of the patient can tolerate the surgical injury, radical surgery was performed on the patient (**Figure 3**).

**Figure 4:**
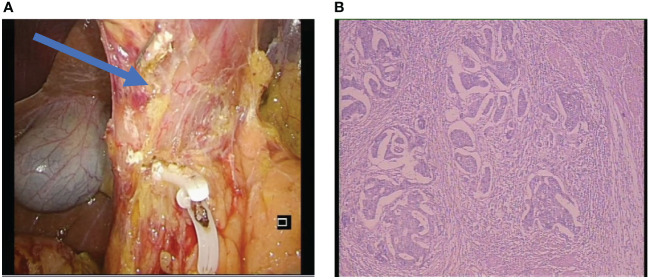
**(A)** The surgical photograph displayed the post-dissection of the sixth group of lymph nodes at the pylorus and revealed the absence of tissue edema. **(B)** Postoperative pathological results, the Becker tumor regression grading showed grade 1b (living tumor cells accounted for less than 10% of the original focus area). The postoperative pathological stage of this patient was ypT2N3aM0.

The radical surgery was ensured, and the pathological examination revealed a significant regression of the tumor (**Figure 4**). The postoperative pathological stage of this patient was ypT2N3aM0. Pathology showed that showed it was microsatellite stable (MSS) gastric cancer. After the operation, the patient was re-examined regularly in our hospital follow the guidelines for follow-up of patients, and there was no obvious abnormality in imaging and tumor marker examination (9). She continues to receive postoperative adjuvant therapy after treatment and there was no obvious recurrence so far.

### 2.5 A summary of nutritional therapy administered in the study

Despite the severe malnutrition and the side effects of chemotherapy drugs, the treatment and surgery proceed smoothly. Nutritional therapy played a key role in maintaining the nutritional, physical and functional status of the patient. The change of PG-SGA scores and the nutritional markers also indicated its efficiency (**Figures 1**, **3**). Considering the potential complications of PN, we administered short-term PN united with oral nutrition support and a tailored dietary plan and no nutrition-related complications were found throughout the treatment.

## 3 Discussions

This is a case of a patient with advanced GC and incomplete pyloric obstruction who benefit from perioperative chemo-immunotherapy (10). It also emphasizes the crucial role of nutritional therapy as an adjunctive treatment. To our knowledge, this is the first report of such a case.

Nutritional support therapy is often imperative during the treatment of advanced GC, particularly as disease progression leads to obstruction. The importance of supportive care including nutritional support, especially for cancer patients who are at high risk of malnutrition have been highlighted by some research (11, 12). Besides, keeping an adequate body-mass index (BMI) and nutritional status can also benefit for advanced patients managed with perioperative therapy, even in second-line regimens as paclitaxel plus ramucirumab (13). In this case, given the risk of DSF after surgery and the high TMB, prioritization of perioperative therapy was deemed necessary (3, 4). According to an international consensus, cachexia should be judged by a combination of BMI, loss of energy stores, and weight loss (14). Although the patient’s BMI is normal, she experienced rapid weight loss and an imbalance in energy intake and expenditure before the therapy. To avoid the deterioration of physical condition, early parenteral nutrition united with oral nutrition support and a tailored dietary plan was consistently implemented throughout the whole treatment. This regimen led to a notable enhancement in patient physical condition and nutritional status, even following the initial cytotoxic phase involving oxaliplatin.

Previous studies have demonstrated that the early implementation of PN can improve physical condition in patients with GC during perioperative chemo-immunotherapy and prolonged the effectiveness of drug responses (15, 16). But we also need to consider the risks of long-term PN (17). Meanwhile, oral nutrition support and a tailored dietary plan, another important component of nutrition support therapy, plays an important supporting role in the treatment of patients with advanced gastric cancer. Thus, based on the pros and cons of above nutritional therapy, our MDT team applied short-term PN combined with oral nutrition support and a tailored dietary plan when the patient had severe gastrointestinal side effects, and stopped PN when the symptoms improved. The absence of nutrition-related complications and the smooth progress of the overall treatment confirmed the efficacy of our protocol.

This case demonstrates a feasible nutritional regimen to improve the physical condition of patients with advanced GC and incomplete pyloric obstruction during perioperative chemo-immunotherapy. However, the specific role of nutritional support therapy in the treatment of gastric cancer is still being explored. At the same time, the oral intake and tolerance with enteral nutritional may vary with the progression of disease (18). Therefore, more research and evidence are needed to confirm if this nutritional regimen can be applied to more patients.

In comparison to traditional chemotherapy regimens, ICIs combined with chemotherapy have shown superior efficacy and fewer side effects as a first-line treatment for selective advanced GC patients (19, 20). Although this regimen has fewer complications and better treatment effects, the limitations and high cost of its application are not suitable for every patient. In recent years, some experiments have confirmed the potential effect of this regimen compared to previous treatments (10, 21). After comparing and summarizing several previous studies, immunotherapy plus chemotherapy showed better treatment effects for gastric cancer patients (22). At the same time, immunotherapy plus chemotherapy can achieve a good prognosis of gastric cancer patients while reducing toxic side effects, which has also been reported in previous case reports (23–25). In our case, the patient suffered from gastrointestinal and myelosuppressive side effects due to intravenous oxaliplatin. However, the patient still achieved PR without the treatment with oxaliplatin. Therefore, for selective patients, cytotoxic drugs may be only needed in the early stages of treatment, and immunotherapy can be the primary option for subsequent stages. However, more clinical data are required to clarify the efficacy of this strategy.

Moreover, current ICIs primarily target specific populations (4). According to the ASCO guidelines, immunotherapy combined with chemotherapy was recommended when CPS is between 1 to 5 (9). In our case, considering that the patient has a CPS score of 1.7 and also has a high risk of DSF and a high TMB, the patient received perioperative chemo-immunotherapy first and achieved favorable response. The postoperative pathological showed it was MSS gastric cancer. In perspective of immunotherapeutic mechanism, an increase in TMB is associated with increased susceptibility of immune system recognition. Increased susceptibility allows specific patients to achieve better outcomes through ICIs (26). Our case reconfirms the possibility that MSS patients may derive benefits from immunotherapy when TMB is elevated.

## 4 Conclusions

This case highlights the benefits of nutrition therapy in perioperative chemo-immunotherapy for advanced GC with incomplete pyloric obstruction. Short-term of PN united with oral nutrition support and a tailored dietary plan can provide more effective support with fewer complications. Meanwhile we also explored combination of immunotherapy, targeted therapy and chemotherapy for new indications. Using fewer chemotherapy and more immunotherapy may benefit patients with advanced GC and incomplete pyloric obstruction, even in MSS. In the future, more clinical evidence is warranted.

## Data availability statement

The original contributions presented in the study are included in the article/Supplementary Material. Further inquiries can be directed to the corresponding authors.

## Ethics statement

The studies involving humans were approved by the Ethics Committee and Institutional Review Boards of the Yantai Yuhuangding Hospital Affiliated to Medical College of Qingdao University. The studies were conducted in accordance with the local legislation and institutional requirements. The participants provided their written informed consent to participate in this study. Written informed consent was obtained from the individual(s) for the publication of any potentially identifiable images or data included in this article.

## Author contributions

MJ: Writing – review & editing, Writing – original draft, Supervision, Methodology, Funding acquisition. ZY: Writing – original draft, Methodology, Investigation, Data curation, Conceptualization. XH: Writing – original draft, Supervision, Methodology, Investigation, Conceptualization. XW: Writing – review & editing, Validation, Supervision, Methodology. ZZ: Writing – review & editing, Validation, Supervision, Methodology. YZ: Writing – review & editing, Validation, Supervision, Methodology, Funding acquisition. XS: Writing – review & editing, Validation, Supervision, Methodology.
